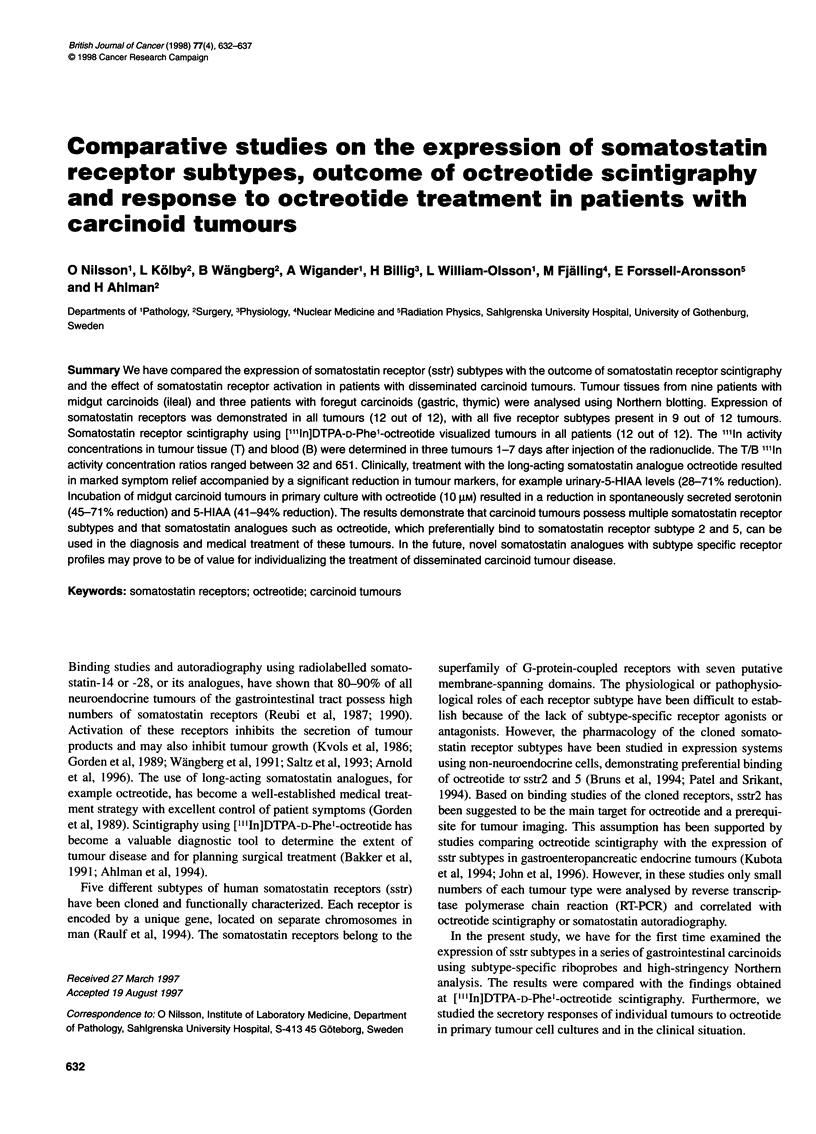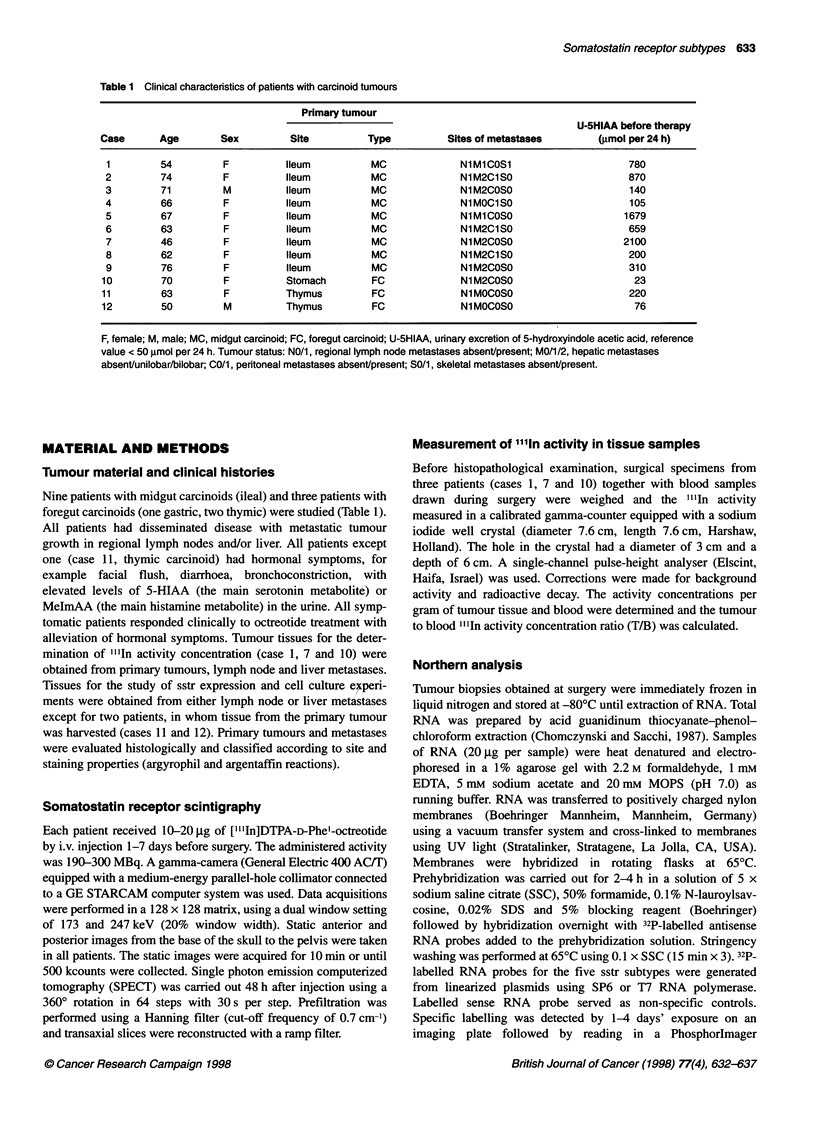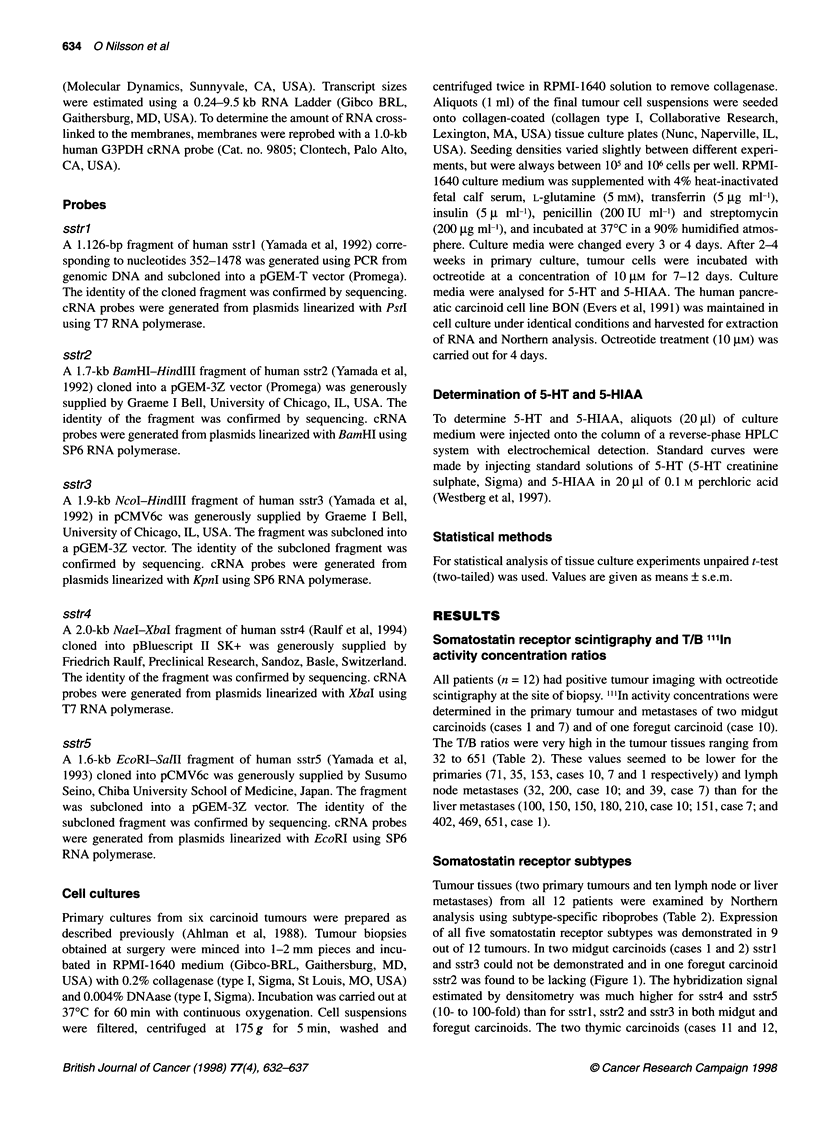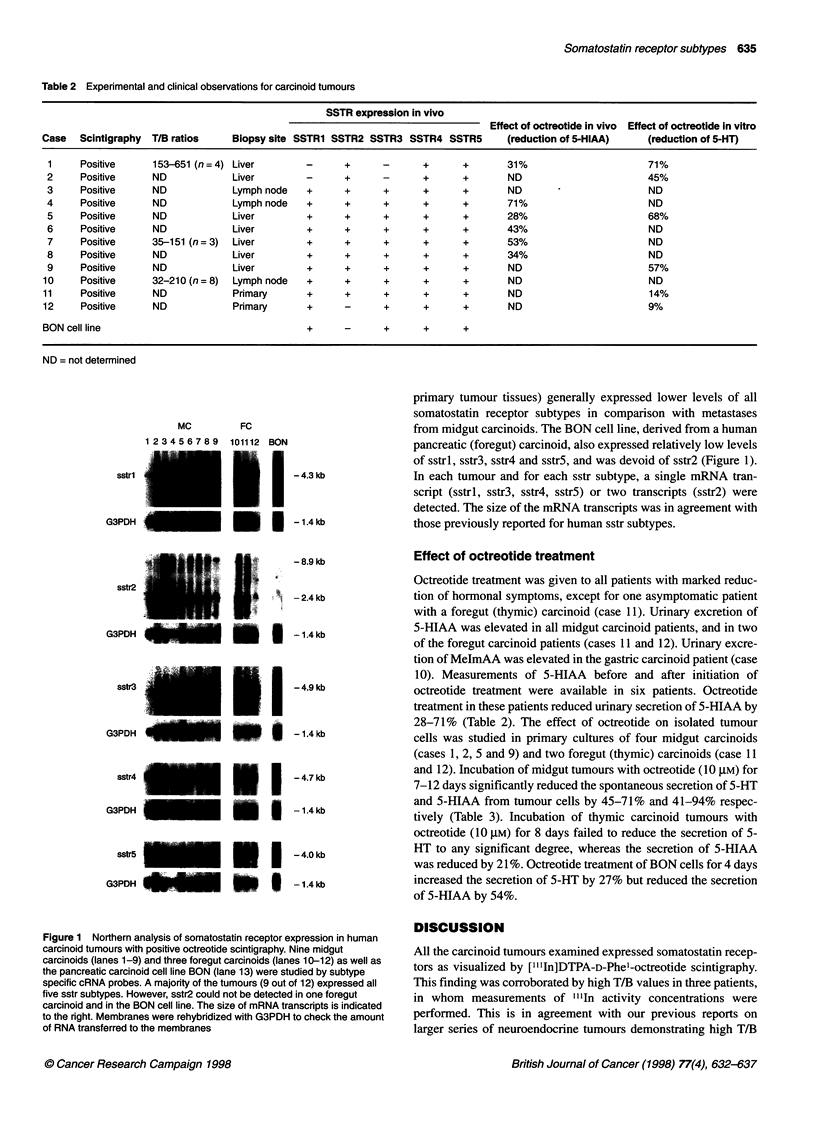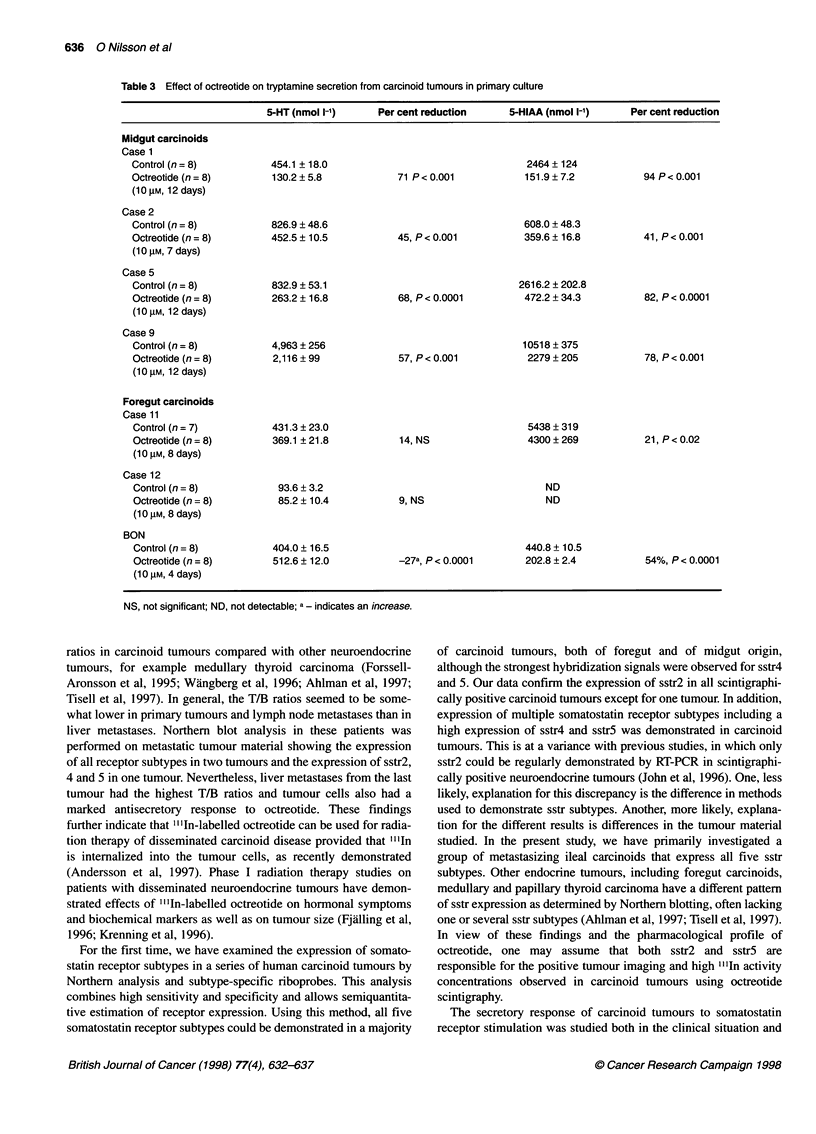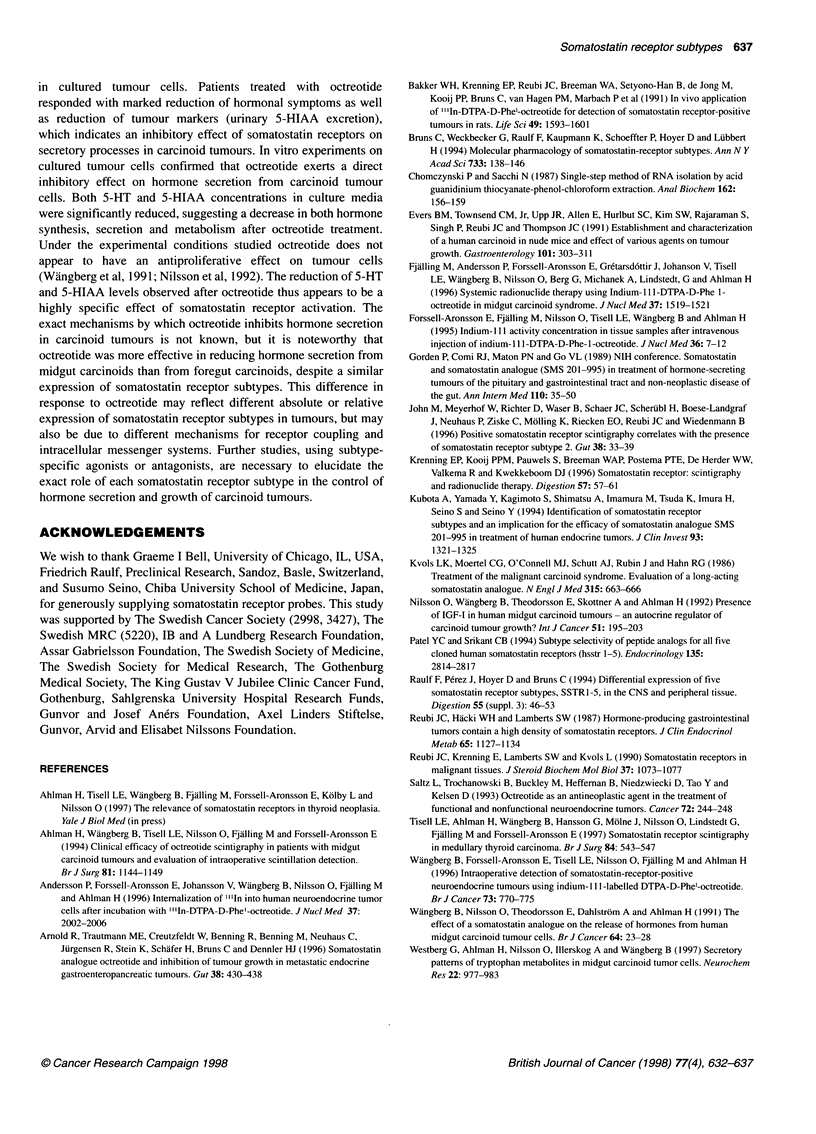# Comparative studies on the expression of somatostatin receptor subtypes, outcome of octreotide scintigraphy and response to octreotide treatment in patients with carcinoid tumours.

**DOI:** 10.1038/bjc.1998.101

**Published:** 1998-02

**Authors:** O. Nilsson, L. KÃ¶lby, B. WÃ¤ngberg, A. Wigander, H. Billig, L. William-Olsson, M. FjÃ¤lling, E. Forssell-Aronsson, H. Ahlman

**Affiliations:** Department of Pathology, Sahlgrenska University Hospital, University of Gothenburg, GÃ¶teborg, Sweden.

## Abstract

**Images:**


British Journal of Cancer (1998) 77(4), 632–637
© 1998 Cancer Research Campaign

### Comparative studies on the expression of somatostatin receptor subtypes, outcome of octreotide scintigraphy and response to octreotide treatment in patients with carcinoid tumours

O Nilsson[1], L Kölby[2], B Wängberg[2], A Wigander[1], H Billig[3], L William-Olsson[1], M Fjälling[4], E Forssell-Aronsson[5] and H Ahlman[2]

Departments of [1]Pathology, [2]Surgery, [3]Physiology, [4]Nuclear Medicine and [5]Radiation Physics, Sahlgrenska University Hospital, University of Gothenburg, Sweden

**Summary** We have compared the expression of somatostatin receptor (sstr) subtypes with the outcome of somatostatin receptor scintigraphy and the effect of somatostatin receptor activation in patients with disseminated carcinoid tumours. Tumour tissues from nine patients with midgut carcinoids (ileal) and three patients with foregut carcinoids (gastric, thymic) were analysed using Northern blotting. Expression of somatostatin receptors was demonstrated in all tumours (12 out of 12), with all five receptor subtypes present in 9 out of 12 tumours. Somatostatin receptor scintigraphy using [$^{111}$In]DTPA-D-Phe$^1$-octreotide visualized tumours in all patients (12 out of 12). The $^{111}$In activity concentrations in tumour tissue (T) and blood (B) were determined in three tumours 1–7 days after injection of the radionuclide. The T/B $^{111}$In activity concentration ratios ranged between 32 and 651. Clinically, treatment with the long-acting somatostatin analogue octreotide resulted in marked symptom relief accompanied by a significant reduction in tumour markers, for example urinary-5-HIAA levels (28–71% reduction). Incubation of midgut carcinoid tumours in primary culture with octreotide (10 μM) resulted in a reduction in spontaneously secreted serotonin (45–71% reduction) and 5-HIAA (41–94% reduction). The results demonstrate that carcinoid tumours possess multiple somatostatin receptor subtypes and that somatostatin analogues such as octreotide, which preferentially bind to somatostatin receptor subtype 2 and 5, can be used in the diagnosis and medical treatment of these tumours. In the future, novel somatostatin analogues with subtype specific receptor profiles may prove to be of value for individualizing the treatment of disseminated carcinoid tumour disease.

**Keywords:** somatostatin receptors; octreotide; carcinoid tumours

Binding studies and autoradiography using radiolabelled somatostatin-14 or -28, or its analogues, have shown that 80–90% of all neuroendocrine tumours of the gastrointestinal tract possess high numbers of somatostatin receptors (Reubi et al, 1987; 1990). Activation of these receptors inhibits the secretion of tumour products and may also inhibit tumour growth (Kvols et al, 1986; Gorden et al, 1989; Wängberg et al, 1991; Saltz et al, 1993; Arnold et al, 1996). The use of long-acting somatostatin analogues, for example octreotide, has become a well-established medical treatment strategy with excellent control of patient symptoms (Gorden et al, 1989). Scintigraphy using [$^{111}$In]DTPA-D-Phe$^1$-octreotide has become a valuable diagnostic tool to determine the extent of tumour disease and for planning surgical treatment (Bakker et al, 1991; Ahlman et al, 1994).

Five different subtypes of human somatostatin receptors (sstr) have been cloned and functionally characterized. Each receptor is encoded by a unique gene, located on separate chromosomes in man (Raulf et al, 1994). The somatostatin receptors belong to the superfamily of G-protein-coupled receptors with seven putative membrane-spanning domains. The physiological or pathophysiological roles of each receptor subtype have been difficult to establish because of the lack of subtype-specific receptor agonists or antagonists. However, the pharmacology of the cloned somatostatin receptor subtypes have been studied in expression systems using non-neuroendocrine cells, demonstrating preferential binding of octreotide to sstr2 and 5 (Bruns et al, 1994; Patel and Srikant, 1994). Based on binding studies of the cloned receptors, sstr2 has been suggested to be the main target for octreotide and a prerequisite for tumour imaging. This assumption has been supported by studies comparing octreotide scintigraphy with the expression of sstr subtypes in gastroenteropancreatic endocrine tumours (Kubota et al, 1994; John et al, 1996). However, in these studies only small numbers of each tumour type were analysed by reverse transcriptase polymerase chain reaction (RT-PCR) and correlated with octreotide scintigraphy or somatostatin autoradiography.

In the present study, we have for the first time examined the expression of sstr subtypes in a series of gastrointestinal carcinoids using subtype-specific riboprobes and high-stringency Northern analysis. The results were compared with the findings obtained at [$^{111}$In]DTPA-D-Phe$^1$-octreotide scintigraphy. Furthermore, we studied the secretory responses of individual tumours to octreotide in primary tumour cell cultures and in the clinical situation.

*Received 27 March 1997*
*Accepted 19 August 1997*

*Correspondence to:* O Nilsson, Institute of Laboratory Medicine, Department of Pathology, Sahlgrenska University Hospital, S-413 45 Göteborg, Sweden

**Table 1** Clinical characteristics of patients with carcinoid tumours

| Case | Age | Sex | Primary tumour Site | Type | Sites of metastases | U-5HIAA before therapy (μmol per 24 h) |
|---|---|---|---|---|---|---|
| 1 | 54 | F | Ileum | MC | N1M1C0S1 | 780 |
| 2 | 74 | F | Ileum | MC | N1M2C1S0 | 870 |
| 3 | 71 | M | Ileum | MC | N1M2C0S0 | 140 |
| 4 | 66 | F | Ileum | MC | N1M0C1S0 | 105 |
| 5 | 67 | F | Ileum | MC | N1M1C0S0 | 1679 |
| 6 | 63 | F | Ileum | MC | N1M2C1S0 | 659 |
| 7 | 46 | F | Ileum | MC | N1M2C0S0 | 2100 |
| 8 | 62 | F | Ileum | MC | N1M2C1S0 | 200 |
| 9 | 76 | F | Ileum | MC | N1M2C0S0 | 310 |
| 10 | 70 | F | Stomach | FC | N1M2C0S0 | 23 |
| 11 | 63 | F | Thymus | FC | N1M0C0S0 | 220 |
| 12 | 50 | M | Thymus | FC | N1M0C0S0 | 76 |

F, female; M, male; MC, midgut carcinoid; FC, foregut carcinoid; U-5HIAA, urinary excretion of 5-hydroxyindole acetic acid, reference value < 50 μmol per 24 h. Tumour status: N0/1, regional lymph node metastases absent/present; M0/1/2, hepatic metastases absent/unilobar/bilobar; C0/1, peritoneal metastases absent/present; S0/1, skeletal metastases absent/present.

### MATERIAL AND METHODS

### Tumour material and clinical histories

Nine patients with midgut carcinoids (ileal) and three patients with foregut carcinoids (one gastric, two thymic) were studied (Table 1). All patients had disseminated disease with metastatic tumour growth in regional lymph nodes and/or liver. All patients except one (case 11, thymic carcinoid) had hormonal symptoms, for example facial flush, diarrhoea, bronchoconstriction, with elevated levels of 5-HIAA (the main serotonin metabolite) or MeImAA (the main histamine metabolite) in the urine. All symptomatic patients responded clinically to octreotide treatment with alleviation of hormonal symptoms. Tumour tissues for the determination of $^{111}$In activity concentration (case 1, 7 and 10) were obtained from primary tumours, lymph node and liver metastases. Tissues for the study of sstr expression and cell culture experiments were obtained from either lymph node or liver metastases except for two patients, in whom tissue from the primary tumour was harvested (cases 11 and 12). Primary tumours and metastases were evaluated histologically and classified according to site and staining properties (argyrophil and argentaffin reactions).

### Somatostatin receptor scintigraphy

Each patient received 10–20 μg of [$^{111}$In]DTPA-D-Phe$^{1}$-octreotide by i.v. injection 1–7 days before surgery. The administered activity was 190–300 MBq. A gamma-camera (General Electric 400 AC/T) equipped with a medium-energy parallel-hole collimator connected to a GE STARCAM computer system was used. Data acquisitions were performed in a 128 × 128 matrix, using a dual window setting of 173 and 247 keV (20% window width). Static anterior and posterior images from the base of the skull to the pelvis were taken in all patients. The static images were acquired for 10 min or until 500 kcounts were collected. Single photon emission computerized tomography (SPECT) was carried out 48 h after injection using a 360° rotation in 64 steps with 30 s per step. Prefiltration was performed using a Hanning filter (cut-off frequency of 0.7 cm$^{-1}$) and transaxial slices were reconstructed with a ramp filter.

### Measurement of $^{111}$In activity in tissue samples

Before histopathological examination, surgical specimens from three patients (cases 1, 7 and 10) together with blood samples drawn during surgery were weighed and the $^{111}$In activity measured in a calibrated gamma-counter equipped with a sodium iodide well crystal (diameter 7.6 cm, length 7.6 cm, Harshaw, Holland). The hole in the crystal had a diameter of 3 cm and a depth of 6 cm. A single-channel pulse-height analyser (Elscint, Haifa, Israel) was used. Corrections were made for background activity and radioactive decay. The activity concentrations per gram of tumour tissue and blood were determined and the tumour to blood $^{111}$In activity concentration ratio (T/B) was calculated.

### Northern analysis

Tumour biopsies obtained at surgery were immediately frozen in liquid nitrogen and stored at –80°C until extraction of RNA. Total RNA was prepared by acid guanidinum thiocyanate–phenol–chloroform extraction (Chomczynski and Sacchi, 1987). Samples of RNA (20 μg per sample) were heat denatured and electrophoresed in a 1% agarose gel with 2.2 M formaldehyde, 1 mM EDTA, 5 mM sodium acetate and 20 mM MOPS (pH 7.0) as running buffer. RNA was transferred to positively charged nylon membranes (Boehringer Mannheim, Mannheim, Germany) using a vacuum transfer system and cross-linked to membranes using UV light (Stratalinker, Stratagene, La Jolla, CA, USA). Membranes were hybridized in rotating flasks at 65°C. Prehybridization was carried out for 2–4 h in a solution of 5 × sodium saline citrate (SSC), 50% formamide, 0.1% N-lauroylsavcosine, 0.02% SDS and 5% blocking reagent (Boehringer) followed by hybridization overnight with $^{32}$P-labelled antisense RNA probes added to the prehybridization solution. Stringency washing was performed at 65°C using 0.1 × SSC (15 min × 3). $^{32}$P-labelled RNA probes for the five sstr subtypes were generated from linearized plasmids using SP6 or T7 RNA polymerase. Labelled sense RNA probe served as non-specific controls. Specific labelling was detected by 1–4 days' exposure on an imaging plate followed by reading in a PhosphorImager

© Cancer Research Campaign 1998

(Molecular Dynamics, Sunnyvale, CA, USA). Transcript sizes were estimated using a 0.24–9.5 kb RNA Ladder (Gibco BRL, Gaithersburg, MD, USA). To determine the amount of RNA cross-linked to the membranes, membranes were reprobed with a 1.0-kb human G3PDH cRNA probe (Cat. no. 9805; Clontech, Palo Alto, CA, USA).

### Probes

### *sstr1*

A 1.126-bp fragment of human sstr1 (Yamada et al, 1992) corresponding to nucleotides 352–1478 was generated using PCR from genomic DNA and subcloned into a pGEM-T vector (Promega). The identity of the cloned fragment was confirmed by sequencing. cRNA probes were generated from plasmids linearized with *Pst*I using T7 RNA polymerase.

### *sstr2*

A 1.7-kb *Bam*HI–*Hin*dIII fragment of human sstr2 (Yamada et al, 1992) cloned into a pGEM-3Z vector (Promega) was generously supplied by Graeme I Bell, University of Chicago, IL, USA. The identity of the fragment was confirmed by sequencing. cRNA probes were generated from plasmids linearized with *Bam*HI using SP6 RNA polymerase.

### *sstr3*

A 1.9-kb *Nco*I–*Hin*dIII fragment of human sstr3 (Yamada et al, 1992) in pCMV6c was generously supplied by Graeme I Bell, University of Chicago, IL, USA. The fragment was subcloned into a pGEM-3Z vector. The identity of the subcloned fragment was confirmed by sequencing. cRNA probes were generated from plasmids linearized with *Kpn*I using SP6 RNA polymerase.

### *sstr4*

A 2.0-kb *Nae*I–*Xba*I fragment of human sstr4 (Raulf et al, 1994) cloned into pBluescript II SK+ was generously supplied by Friedrich Raulf, Preclinical Research, Sandoz, Basle, Switzerland. The identity of the fragment was confirmed by sequencing. cRNA probes were generated from plasmids linearized with *Xba*I using T7 RNA polymerase.

### *sstr5*

A 1.6-kb *Eco*RI–*Sal*II fragment of human sstr5 (Yamada et al, 1993) cloned into pCMV6c was generously supplied by Susumo Seino, Chiba University School of Medicine, Japan. The fragment was subcloned into a pGEM-3Z vector. The identity of the subcloned fragment was confirmed by sequencing. cRNA probes were generated from plasmids linearized with *Eco*RI using SP6 RNA polymerase.

### Cell cultures

Primary cultures from six carcinoid tumours were prepared as described previously (Ahlman et al, 1988). Tumour biopsies obtained at surgery were minced into 1–2 mm pieces and incubated in RPMI-1640 medium (Gibco-BRL, Gaithersburg, MD, USA) with 0.2% collagenase (type I, Sigma, St Louis, MO, USA) and 0.004% DNAase (type I, Sigma). Incubation was carried out at 37°C for 60 min with continuous oxygenation. Cell suspensions were filtered, centrifuged at 175 *g* for 5 min, washed and centrifuged twice in RPMI-1640 solution to remove collagenase. Aliquots (1 ml) of the final tumour cell suspensions were seeded onto collagen-coated (collagen type I, Collaborative Research, Lexington, MA, USA) tissue culture plates (Nunc, Naperville, IL, USA). Seeding densities varied slightly between different experiments, but were always between $10^5$ and $10^6$ cells per well. RPMI-1640 culture medium was supplemented with 4% heat-inactivated fetal calf serum, L-glutamine (5 mM), transferrin (5 μg $ml^{-1}$), insulin (5 μ $ml^{-1}$), penicillin (200 IU $ml^{-1}$) and streptomycin (200 μg $ml^{-1}$), and incubated at 37°C in a 90% humidified atmosphere. Culture media were changed every 3 or 4 days. After 2–4 weeks in primary culture, tumour cells were incubated with octreotide at a concentration of 10 μM for 7–12 days. Culture media were analysed for 5-HT and 5-HIAA. The human pancreatic carcinoid cell line BON (Evers et al, 1991) was maintained in cell culture under identical conditions and harvested for extraction of RNA and Northern analysis. Octreotide treatment (10 μM) was carried out for 4 days.

### Determination of 5-HT and 5-HIAA

To determine 5-HT and 5-HIAA, aliquots (20 μl) of culture medium were injected onto the column of a reverse-phase HPLC system with electrochemical detection. Standard curves were made by injecting standard solutions of 5-HT (5-HT creatinine sulphate, Sigma) and 5-HIAA in 20 μl of 0.1 M perchloric acid (Westberg et al, 1997).

### Statistical methods

For statistical analysis of tissue culture experiments unpaired *t*-test (two-tailed) was used. Values are given as means ± s.e.m.

### RESULTS

### Somatostatin receptor scintigraphy and T/B $^{111}$In activity concentration ratios

All patients ($n$ = 12) had positive tumour imaging with octreotide scintigraphy at the site of biopsy. $^{111}$In activity concentrations were determined in the primary tumour and metastases of two midgut carcinoids (cases 1 and 7) and of one foregut carcinoid (case 10). The T/B ratios were very high in the tumour tissues ranging from 32 to 651 (Table 2). These values seemed to be lower for the primaries (71, 35, 153, cases 10, 7 and 1 respectively) and lymph node metastases (32, 200, case 10; and 39, case 7) than for the liver metastases (100, 150, 150, 180, 210, case 10; 151, case 7; and 402, 469, 651, case 1).

### Somatostatin receptor subtypes

Tumour tissues (two primary tumours and ten lymph node or liver metastases) from all 12 patients were examined by Northern analysis using subtype-specific riboprobes (Table 2). Expression of all five somatostatin receptor subtypes was demonstrated in 9 out of 12 tumours. In two midgut carcinoids (cases 1 and 2) sstr1 and sstr3 could not be demonstrated and in one foregut carcinoid sstr2 was found to be lacking (Figure 1). The hybridization signal estimated by densitometry was much higher for sstr4 and sstr5 (10- to 100-fold) than for sstr1, sstr2 and sstr3 in both midgut and foregut carcinoids. The two thymic carcinoids (cases 11 and 12,

© Cancer Research Campaign 1998

**Table 2** Experimental and clinical observations for carcinoid tumours

| Case | Scintigraphy | T/B ratios | Biopsy site | SSTR expression in vivo | | | | | Effect of octreotide in vivo (reduction of 5-HIAA) | Effect of octreotide in vitro (reduction of 5-HT) |
|---|---|---|---|---|---|---|---|---|---|---|
| | | | | SSTR1 | SSTR2 | SSTR3 | SSTR4 | SSTR5 | | |
| 1 | Positive | 153–651 (*n* = 4) | Liver | – | + | – | + | + | 31% | 71% |
| 2 | Positive | ND | Liver | – | + | – | + | + | ND | 45% |
| 3 | Positive | ND | Lymph node | + | + | + | + | + | ND | ND |
| 4 | Positive | ND | Lymph node | + | + | + | + | + | 71% | ND |
| 5 | Positive | ND | Liver | + | + | + | + | + | 28% | 68% |
| 6 | Positive | ND | Liver | + | + | + | + | + | 43% | ND |
| 7 | Positive | 35–151 (*n* = 3) | Liver | + | + | + | + | + | 53% | ND |
| 8 | Positive | ND | Liver | + | + | + | + | + | 34% | ND |
| 9 | Positive | ND | Liver | + | + | + | + | + | ND | 57% |
| 10 | Positive | 32–210 (*n* = 8) | Lymph node | + | + | + | + | + | ND | ND |
| 11 | Positive | ND | Primary | + | + | + | + | + | ND | 14% |
| 12 | Positive | ND | Primary | + | – | + | + | + | ND | 9% |
| BON cell line | | | | + | – | + | + | + | | |

ND = not determined

**Figure 1** Northern analysis of somatostatin receptor expression in human carcinoid tumours with positive octreotide scintigraphy. Nine midgut carcinoids (lanes 1–9) and three foregut carcinoids (lanes 10–12) as well as the pancreatic carcinoid cell line BON (lane 13) were studied by subtype specific cRNA probes. A majority of the tumours (9 out of 12) expressed all five sstr subtypes. However, sstr2 could not be detected in one foregut carcinoid and in the BON cell line. The size of mRNA transcripts is indicated to the right. Membranes were rehybridized with G3PDH to check the amount of RNA transferred to the membranes primary tumour tissues) generally expressed lower levels of all somatostatin receptor subtypes in comparison with metastases from midgut carcinoids. The BON cell line, derived from a human pancreatic (foregut) carcinoid, also expressed relatively low levels of sstr1, sstr3, sstr4 and sstr5, and was devoid of sstr2 (Figure 1). In each tumour and for each sstr subtype, a single mRNA transcript (sstr1, sstr3, sstr4, sstr5) or two transcripts (sstr2) were detected. The size of the mRNA transcripts was in agreement with those previously reported for human sstr subtypes.

### Effect of octreotide treatment

Octreotide treatment was given to all patients with marked reduction of hormonal symptoms, except for one asymptomatic patient with a foregut (thymic) carcinoid (case 11). Urinary excretion of 5-HIAA was elevated in all midgut carcinoid patients, and in two of the foregut carcinoid patients (cases 11 and 12). Urinary excretion of MeImAA was elevated in the gastric carcinoid patient (case 10). Measurements of 5-HIAA before and after initiation of octreotide treatment were available in six patients. Octreotide treatment in these patients reduced urinary secretion of 5-HIAA by 28–71% (Table 2). The effect of octreotide on isolated tumour cells was studied in primary cultures of four midgut carcinoids (cases 1, 2, 5 and 9) and two foregut (thymic) carcinoids (case 11 and 12). Incubation of midgut tumours with octreotide (10 μM) for 7–12 days significantly reduced the spontaneous secretion of 5-HT and 5-HIAA from tumour cells by 45–71% and 41–94% respectively (Table 3). Incubation of thymic carcinoid tumours with octreotide (10 μM) for 8 days failed to reduce the secretion of 5-HT to any significant degree, whereas the secretion of 5-HIAA was reduced by 21%. Octreotide treatment of BON cells for 4 days increased the secretion of 5-HT by 27% but reduced the secretion of 5-HIAA by 54%.

### DISCUSSION

All the carcinoid tumours examined expressed somatostatin receptors as visualized by [$^{111}$In]DTPA-D-Phe$^{1}$-octreotide scintigraphy. This finding was corroborated by high T/B values in three patients, in whom measurements of $^{111}$In activity concentrations were performed. This is in agreement with our previous reports on larger series of neuroendocrine tumours demonstrating high T/B

**Table 3** Effect of octreotide on tryptamine secretion from carcinoid tumours in primary culture

| | 5-HT (nmol l$^{-1}$) | Per cent reduction | 5-HIAA (nmol l$^{-1}$) | Per cent reduction |
|---|---|---|---|---|
| **Midgut carcinoids** | | | | |
| Case 1 | | | | |
| Control ($n$ = 8) | 454.1 ± 18.0 | | 2464 ± 124 | |
| Octreotide ($n$ = 8) | 130.2 ± 5.8 | 71 $P$ < 0.001 | 151.9 ± 7.2 | 94 $P$ < 0.001 |
| (10 µM, 12 days) | | | | |
| Case 2 | | | | |
| Control ($n$ = 8) | 826.9 ± 48.6 | | 608.0 ± 48.3 | |
| Octreotide ($n$ = 8) | 452.5 ± 10.5 | 45, $P$ < 0.001 | 359.6 ± 16.8 | 41, $P$ < 0.001 |
| (10 µM, 7 days) | | | | |
| Case 5 | | | | |
| Control ($n$ = 8) | 832.9 ± 53.1 | | 2616.2 ± 202.8 | |
| Octreotide ($n$ = 8) | 263.2 ± 16.8 | 68, $P$ < 0.0001 | 472.2 ± 34.3 | 82, $P$ < 0.0001 |
| (10 µM, 12 days) | | | | |
| Case 9 | | | | |
| Control ($n$ = 8) | 4,963 ± 256 | | 10518 ± 375 | |
| Octreotide ($n$ = 8) | 2,116 ± 99 | 57, $P$ < 0.001 | 2279 ± 205 | 78, $P$ < 0.001 |
| (10 µM, 12 days) | | | | |
| **Foregut carcinoids** | | | | |
| Case 11 | | | | |
| Control ($n$ = 7) | 431.3 ± 23.0 | | 5438 ± 319 | |
| Octreotide ($n$ = 8) | 369.1 ± 21.8 | 14, NS | 4300 ± 269 | 21, $P$ < 0.02 |
| (10 µM, 8 days) | | | | |
| Case 12 | | | | |
| Control ($n$ = 8) | 93.6 ± 3.2 | | ND | |
| Octreotide ($n$ = 8) | 85.2 ± 10.4 | 9, NS | ND | |
| (10 µM, 8 days) | | | | |
| BON | | | | |
| Control ($n$ = 8) | 404.0 ± 16.5 | | 440.8 ± 10.5 | |
| Octreotide ($n$ = 8) | 512.6 ± 12.0 | −27[a], $P$ < 0.0001 | 202.8 ± 2.4 | 54%, $P$ < 0.0001 |
| (10 µM, 4 days) | | | | |

NS, not significant; ND, not detectable; [a] – indicates an *increase*.

ratios in carcinoid tumours compared with other neuroendocrine tumours, for example medullary thyroid carcinoma (Forssell-Aronsson et al, 1995; Wängberg et al, 1996; Ahlman et al, 1997; Tisell et al, 1997). In general, the T/B ratios seemed to be somewhat lower in primary tumours and lymph node metastases than in liver metastases. Northern blot analysis in these patients was performed on metastatic tumour material showing the expression of all receptor subtypes in two tumours and the expression of sstr2, 4 and 5 in one tumour. Nevertheless, liver metastases from the last tumour had the highest T/B ratios and tumour cells also had a marked antisecretory response to octreotide. These findings further indicate that $^{111}$In-labelled octreotide can be used for radiation therapy of disseminated carcinoid disease provided that $^{111}$In is internalized into the tumour cells, as recently demonstrated (Andersson et al, 1997). Phase I radiation therapy studies on patients with disseminated neuroendocrine tumours have demonstrated effects of $^{111}$In-labelled octreotide on hormonal symptoms and biochemical markers as well as on tumour size (Fjälling et al, 1996; Krenning et al, 1996).

For the first time, we have examined the expression of somatostatin receptor subtypes in a series of human carcinoid tumours by Northern analysis and subtype-specific riboprobes. This analysis combines high sensitivity and specificity and allows semiquantitative estimation of receptor expression. Using this method, all five somatostatin receptor subtypes could be demonstrated in a majority of carcinoid tumours, both of foregut and of midgut origin, although the strongest hybridization signals were observed for sstr4 and 5. Our data confirm the expression of sstr2 in all scintigraphically positive carcinoid tumours except for one tumour. In addition, expression of multiple somatostatin receptor subtypes including a high expression of sstr4 and sstr5 was demonstrated in carcinoid tumours. This is at a variance with previous studies, in which only sstr2 could be regularly demonstrated by RT-PCR in scintigraphically positive neuroendocrine tumours (John et al, 1996). One, less likely, explanation for this discrepancy is the difference in methods used to demonstrate sstr subtypes. Another, more likely, explanation for the different results is differences in the tumour material studied. In the present study, we have primarily investigated a group of metastasizing ileal carcinoids that express all five sstr subtypes. Other endocrine tumours, including foregut carcinoids, medullary and papillary thyroid carcinoma have a different pattern of sstr expression as determined by Northern blotting, often lacking one or several sstr subtypes (Ahlman et al, 1997; Tisell et al, 1997). In view of these findings and the pharmacological profile of octreotide, one may assume that both sstr2 and sstr5 are responsible for the positive tumour imaging and high $^{111}$In activity concentrations observed in carcinoid tumours using octreotide scintigraphy.

The secretory response of carcinoid tumours to somatostatin receptor stimulation was studied both in the clinical situation and

 © Cancer Research Campaign 1998

in cultured tumour cells. Patients treated with octreotide responded with marked reduction of hormonal symptoms as well as reduction of tumour markers (urinary 5-HIAA excretion), which indicates an inhibitory effect of somatostatin receptors on secretory processes in carcinoid tumours. In vitro experiments on cultured tumour cells confirmed that octreotide exerts a direct inhibitory effect on hormone secretion from carcinoid tumour cells. Both 5-HT and 5-HIAA concentrations in culture media were significantly reduced, suggesting a decrease in both hormone synthesis, secretion and metabolism after octreotide treatment. Under the experimental conditions studied octreotide does not appear to have an antiproliferative effect on tumour cells (Wängberg et al, 1991; Nilsson et al, 1992). The reduction of 5-HT and 5-HIAA levels observed after octreotide thus appears to be a highly specific effect of somatostatin receptor activation. The exact mechanisms by which octreotide inhibits hormone secretion in carcinoid tumours is not known, but it is noteworthy that octreotide was more effective in reducing hormone secretion from midgut carcinoids than from foregut carcinoids, despite a similar expression of somatostatin receptor subtypes. This difference in response to octreotide may reflect different absolute or relative expression of somatostatin receptor subtypes in tumours, but may also be due to different mechanisms for receptor coupling and intracellular messenger systems. Further studies, using subtype-specific agonists or antagonists, are necessary to elucidate the exact role of each somatostatin receptor subtype in the control of hormone secretion and growth of carcinoid tumours.

### ACKNOWLEDGEMENTS

We wish to thank Graeme I Bell, University of Chicago, IL, USA, Friedrich Raulf, Preclinical Research, Sandoz, Basle, Switzerland, and Susumo Seino, Chiba University School of Medicine, Japan, for generously supplying somatostatin receptor probes. This study was supported by The Swedish Cancer Society (2998, 3427), The Swedish MRC (5220), IB and A Lundberg Research Foundation, Assar Gabrielsson Foundation, The Swedish Society of Medicine, The Swedish Society for Medical Research, The Gothenburg Medical Society, The King Gustav V Jubilee Clinic Cancer Fund, Gothenburg, Sahlgrenska University Hospital Research Funds, Gunvor and Josef Anérs Foundation, Axel Linders Stiftelse, Gunvor, Arvid and Elisabet Nilssons Foundation.

### REFERENCES

Ahlman H, Tisell LE, Wängberg B, Fjälling M, Forssell-Aronsson E, Kölby L and Nilsson O (1997) The relevance of somatostatin receptors in thyroid neoplasia. *Yale J Biol Med* (in press)

Ahlman H, Wängberg B, Tisell LE, Nilsson O, Fjälling M and Forssell-Aronsson E (1994) Clinical efficacy of octreotide scintigraphy in patients with midgut carcinoid tumours and evaluation of intraoperative scintillation detection. *Br J Surg* **81:** 1144–1149

Andersson P, Forssell-Aronsson E, Johansson V, Wängberg B, Nilsson O, Fjälling M and Ahlman H (1996) Internalization of $^{111}$In into human neuroendocrine tumor cells after incubation with $^{111}$In-DTPA-D-Phe$^{1}$-octreotide. *J Nucl Med* **37:** 2002–2006

Arnold R, Trautmann ME, Creutzfeldt W, Benning R, Benning M, Neuhaus C, Jürgensen R, Stein K, Schäfer H, Bruns C and Dennler HJ (1996) Somatostatin analogue octreotide and inhibition of tumour growth in metastatic endocrine gastroenteropancreatic tumours. *Gut* **38:** 430–438

Bakker WH, Krenning EP, Reubi JC, Breeman WA, Setyono-Han B, de Jong M, Kooij PP, Bruns C, van Hagen PM, Marbach P et al (1991) In vivo application of $^{111}$In-DTPA-D-Phe$^{1}$-octreotide for detection of somatostatin receptor-positive tumours in rats. *Life Sci* **49:** 1593–1601

Bruns C, Weckbecker G, Raulf F, Kaupmann K, Schoeffter P, Hoyer D and Lübbert H (1994) Molecular pharmacology of somatostatin-receptor subtypes. *Ann N Y Acad Sci* **733:** 138–146

Chomczynski P and Sacchi N (1987) Single-step method of RNA isolation by acid guanidinium thiocyanate-phenol-chloroform extraction. *Anal Biochem* **162:** 156–159

Evers BM, Townsend CM, Jr, Upp JR, Allen E, Hurlbut SC, Kim SW, Rajaraman S, Singh P, Reubi JC and Thompson JC (1991) Establishment and characterization of a human carcinoid in nude mice and effect of various agents on tumour growth. *Gastroenterology* **101:** 303–311

Fjälling M, Andersson P, Forssell-Aronsson E, Grétarsdóttir J, Johanson V, Tisell LE, Wängberg B, Nilsson O, Berg G, Michanek A, Lindstedt, G and Ahlman H (1996) Systemic radionuclide therapy using Indium-111-DTPA-D-Phe 1-octreotide in midgut carcinoid syndrome. *J Nucl Med* **37:** 1519–1521

Forssell-Aronsson E, Fjälling M, Nilsson O, Tisell LE, Wängberg B and Ahlman H (1995) Indium-111 activity concentration in tissue samples after intravenous injection of indium-111-DTPA-D-Phe-1-octreotide. *J Nucl Med* **36:** 7–12

Gorden P, Comi RJ, Maton PN and Go VL (1989) NIH conference. Somatostatin and somatostatin analogue (SMS 201–995) in treatment of hormone-secreting tumours of the pituitary and gastrointestinal tract and non-neoplastic disease of the gut. *Ann Intern Med* **110:** 35–50

John M, Meyerhof W, Richter D, Waser B, Schaer JC, Scherübl H, Boese-Landgraf J, Neuhaus P, Ziske C, Mölling K, Riecken EO, Reubi JC and Wiedenmann B (1996) Positive somatostatin receptor scintigraphy correlates with the presence of somatostatin receptor subtype 2. *Gut* **38:** 33–39

Krenning EP, Kooij PPM, Pauwels S, Breeman WAP, Postema PTE, De Herder WW, Valkema R and Kwekkeboom DJ (1996) Somatostatin receptor: scintigraphy and radionuclide therapy. *Digestion* **57:** 57–61

Kubota A, Yamada Y, Kagimoto S, Shimatsu A, Imamura M, Tsuda K, Imura H, Seino S and Seino Y (1994) Identification of somatostatin receptor subtypes and an implication for the efficacy of somatostatin analogue SMS 201–995 in treatment of human endocrine tumors. *J Clin Invest* **93:** 1321–1325

Kvols LK, Moertel CG, O'Connell MJ, Schutt AJ, Rubin J and Hahn RG (1986) Treatment of the malignant carcinoid syndrome. Evaluation of a long-acting somatostatin analogue. *N Engl J Med* **315:** 663–666

Nilsson O, Wängberg B, Theodorsson E, Skottner A and Ahlman H (1992) Presence of IGF-I in human midgut carcinoid tumours – an autocrine regulator of carcinoid tumour growth? *Int J Cancer* **51:** 195–203

Patel YC and Srikant CB (1994) Subtype selectivity of peptide analogs for all five cloned human somatostatin receptors (hsstr 1–5). *Endocrinology* **135:** 2814–2817

Raulf F, Pérez J, Hoyer D and Bruns C (1994) Differential expression of five somatostatin receptor subtypes, SSTR1-5, in the CNS and peripheral tissue. *Digestion* **55** (suppl. 3): 46–53

Reubi JC, Häcki WH and Lamberts SW (1987) Hormone-producing gastrointestinal tumors contain a high density of somatostatin receptors. *J Clin Endocrinol Metab* **65:** 1127–1134

Reubi JC, Krenning E, Lamberts SW and Kvols L (1990) Somatostatin receptors in malignant tissues. *J Steroid Biochem Mol Biol* **37:** 1073–1077

Saltz L, Trochanowski B, Buckley M, Heffernan B, Niedzwiecki D, Tao Y and Kelsen D (1993) Octreotide as an antineoplastic agent in the treatment of functional and nonfunctional neuroendocrine tumors. *Cancer* **72:** 244–248

Tisell LE, Ahlman H, Wängberg B, Hansson G, Mölne J, Nilsson O, Lindstedt G, Fjälling M and Forssell-Aronsson E (1997) Somatostatin receptor scintigraphy in medullary thyroid carcinoma. *Br J Surg* **84:** 543–547

Wängberg B, Forssell-Aronsson E, Tisell LE, Nilsson O, Fjälling M and Ahlman H (1996) Intraoperative detection of somatostatin-receptor-positive neuroendocrine tumours using indium-111-labelled DTPA-D-Phe$^{1}$-octreotide. *Br J Cancer* **73:** 770–775

Wängberg B, Nilsson O, Theodorsson E, Dahlström A and Ahlman H (1991) The effect of a somatostatin analogue on the release of hormones from human midgut carcinoid tumour cells. *Br J Cancer* **64:** 23–28

Westberg G, Ahlman H, Nilsson O, Illerskog A and Wängberg B (1997) Secretory patterns of tryptophan metabolites in midgut carcinoid tumor cells. *Neurochem Res* **22:** 977–983

© Cancer Research Campaign 1998